# Chinese Patent Medicine Nei-Xiao-Pian Shrinks Thyroid Nodules by Regulating Proliferation and Apoptosis of Cells *via* Inhibiting the IL-6/JAK2/STAT3 Signaling Pathway

**DOI:** 10.2174/0118715303380988250627113427

**Published:** 2025-07-09

**Authors:** Yu-Ting Zhao, Yang Yang, Wen-Ting Mei, Ting Chen, Wei Wei, Yu-Chen Wu, Xiao Yu, Fei Huang

**Affiliations:** 1 Department of Endocrinology, The Suzhou Affiliated Hospital of Nanjing University of Chinese Medicine, 18 Yangsu Road, Suzhou 215000, China;; 2 Laboratory of Cancer Molecular Genetics, Medical College of Soochow University, 199 Ren-Ai Road, Dushu Lake Campus, Suzhou Industrial Park, Suzhou 215123, P.R. China

**Keywords:** Thyroid nodules, Chinese patent medicine, Nei-Xiao-Pian (NXP), cell proliferation and apoptosis, IL-6/JAK2/STAT3 signaling pathway

## Abstract

**Background:**

Thyroid nodules (TN) are distinct lesions within the thyroid gland that can cause compression symptoms, disrupt thyroid function, and impact aesthetics and daily life. Nei-Xiao-Pian (NXP), a traditional Chinese patent medicine developed by Suzhou Hospital of Traditional Chinese Medicine, has been effectively used in clinical treatment for TN. Due to the complex nature of traditional Chinese medicine (TCM), limited clinical research exists on NXP, and its precise mechanisms of action remain largely unknown.

**Methods:**

In this study, we investigated the effects of NXP on human tumorous thyroid cells and papillary thyroid carcinoma (PTC) cells. Cells were treated with animal serum containing different concentrations of NXP. Cell viability, cell cycle progression, and apoptosis were assessed using the CCK-8 assay and flow cytometry. A literature review was also conducted to understand the physiological and pathological mechanisms underlying TN. Furthermore, we examined the involvement of the IL6/JAK2/STAT3 signaling pathway in the action of NXP by measuring the protein expression levels of IL-6, JAK2, and STAT3 in PTC cells using western blotting.

**Results:**

The present study showed that serum with a specific concentration of NXP reduced the viability of both tumorous thyroid cells and PTC cells, inhibited the proliferation of tumorous thyroid cells, and induced apoptosis. While NXP-containing serum did not significantly affect the cell cycle in PTC cells, it induced apoptosis in a dose-dependent manner. Additionally, NXP-containing serum dose-dependently suppressed the protein expressions of IL-6, JAK2, and STAT3 in PTC cells.

**Discussion:**

This study shows NXP-containing serum reduces thyroid tumor and PTC cell viability, inhibits proliferation, induces apoptosis (dose-dependent in PTC). It suppresses IL-6/JAK2/STAT3 in PTC, suggesting anti-tumor potential via this pathway, needing in vivo validation.

**Conclusion:**

Our findings suggest that NXP may exert its therapeutic effects on TN by targeting the IL6/JAK2/STAT3 signaling pathway, thereby slowing disease progression. These results highlight a potential mechanism through which NXP could contribute to tumor prevention and treatment, offering a promising direction for future research.

## 1 INTRODUCTION

The thyroid gland, consisting of two connected lobes, is one of the largest endocrine glands in the human body, weighing 20-30 g in adults. Thyroid lesions are often found on the gland, with a prevalence of 4%-7%. Benign lesions include simple or hemorrhagic cysts, colloid nodules, and thyroid adenomas. These lesions may be inactive or active in producing thyroid hormones. In the case of being active, they may be referred to as toxic thyroid adenomas. Patients with thyroid adenomas are usually asymptomatic. However, biochemical and clinical hyperthyroidism can be caused by a toxic adenoma, defined as an autonomously functioning thyroid nodule (AFTN) [1]. Thyroid nodules (TN) are a common endocrine condition frequently encountered in medical practice, arising from the abnormal growth and repair of thyroid cells due to various causes, leading to the formation of scattered lesions. TN can be classified into benign types, such as thyroid adenomas and nodular goiters, as well as malignant forms like thyroid cancer, all of which generally develop slowly and subtly [2]. While most individuals with TN remain asymptomatic, some may experience discomfort in the front of their neck. In its early stages, thyroid cancer often lacks clear symptoms, which can delay detection. As the disease progresses, nodules may enlarge rapidly or spread to nearby vital structures, causing multiple complications. This progression complicates treatment and can significantly impact the patient’s physical and mental health, posing a considerable burden on families and society [3, 4].

The widespread use of high-resolution ultrasound technology and increased awareness of health screenings have contributed to a higher detection rate of TN [5]. The American Thyroid Association reported that high-resolution ultrasound can detect thyroid nodules in 19%-68% of randomly selected individuals, with higher frequencies in women and the elderly [6]. Based on the GLOBOCAN 2020 database of cancer incidence and mortality by the WHO International Agency for Research on Cancer, thyroid cancer has the ninth highest cancer incidence worldwide [7]. Given the large number of affected individuals, the development of effective and multifaceted treatment strategies is crucial. Current Western medical guidelines generally recommend a conservative approach, emphasizing regular monitoring and careful observation of nodule changes. However, clinical practice often reveals patient anxiety and related symptoms, highlighting a preference for more proactive treatment. Thus, we propose integrating the comprehensive and individualized approach of traditional Chinese medicine, focusing on early intervention and syndrome differentiation, to enhance the treatment options for TN.

Chinese medicine formulas typically consist of a blend of herbs that work synergistically through multiple therapeutic pathways to achieve their effects. Nei-Xiao-Pian (NXP), a proprietary treatment developed by Suzhou Hospital of Traditional Chinese Medicine, is composed of five main ingredients: *mylabris phalerata (ban-mao), scolopendra (wu-gong), lumbricus terrestris (di-long), processed bombyx batryticatus (zhi-jiang-can)*, and *pangolin scales (chuan-shan-jia-pian)*. This formula has long been used to treat benign thyroid nodules. Previous clinical trials conducted in China have shown that NXP can effectively reduce benign nodules and alleviate related symptoms, such as excess phlegm, tightness in the chest, and flank pain, thus improving the patients’ propensity for developing tumors. The treatment has demonstrated significant effectiveness and a high safety profile, with no reported adverse effects on liver, kidney function, or gastrointestinal health. While global research into thyroid nodules (TN) has identified numerous risk factors, the exact mechanisms underlying TN development remain somewhat unclear. Studies commonly point to chronic low-grade inflammation [8], insulin resistance [9], abnormal leptin secretion [10], genetic predisposition, and exposure to ionizing radiation. These factors disrupt the balance between thyroid cell growth and death, which is crucial in TN pathogenesis [11]. Recent studies have shed light on the IL-6/JAK2/STAT3 signaling pathway, known for its role in cell growth, differentiation, immune response, and vascular development, and its aberrant activation in various cancers [12, 13]. This pathway’s excessive activation is increasingly recognized as central to thyroid cancer’s development and progression [14, 15], positioning it as a promising target for therapeutic intervention [16, 17].

The Nei-Xiao-Pian (NXP) formula, with its complex blend of traditional Chinese medicinal ingredients, offers an intriguing subject for investigation in the treatment of TN. Building on initial clinical observations, our study utilizes thyroid tumor and papillary thyroid carcinoma (PTC) cell models to explore how NXP-enriched serum at various concentrations affects cell behavior, including growth, proliferation, and apoptosis. Additionally, we explore the potential involvement of the IL-6/JAK2/STAT3 signaling pathway in NXP’s mechanism of action. Our aim is to provide experimental evidence and new insights into the diagnosis and treatment of TN, furthering the understanding of NXP’s therapeutic mechanisms.

## 2 MATERIALS AND METHODS

### 2.1 Composition and Preparation of NXP

NXP, a proprietary Chinese medicine, is chiefly formulated from five insect-derived ingredients: *mylabris phalerata (ban-mao), scolopendra (wu-gong), lumbricus terrestris (Di-long), processed bombyx batryticatus (zhi-jiang-can)*, and *pangolin scales (chuan-shan-jia-pian)*. This medicine was acquired from the Chinese Medicine Pharmacy at Suzhou Hospital of Traditional Chinese Medicine, where it was prepared according to the hospital’s standardized methods. The preparation is identified by the Batch Number: SuMed Approval Z04001567. Specimens of each ingredient are stored at the herbarium center of the Suzhou Chinese Traditional Medicine Hospital for reference.

### 2.2 Animal Source and Housing Conditions

This study employed male rats as subjects due to the known influence of estrogen on thyroid cell functions; thus, male rats were selected. We sourced twenty male rats, weighing an average of 403 ± 26g (15-week-old), from the animal center of Soochow University. These rats were housed under controlled conditions: groups of five per cage, in a standard environment with temperatures ranging from 22-25°C, humidity levels of 40%-70%, proper ventilation, and unrestricted access to water and food. The animals were evenly divided into two experimental groups for oral administration: a low-dose group and a medium-dose group, with ten rats in each. The creation of this animal model, along with the behavioral and biochemical evaluations performed, adhered strictly to the “Public Health Service Policy On Humane Care and Use of Laboratory Animals” (2015 revision) set forth by the U.S. Department of Health and Human Services, with a concerted effort to minimize the use of experimental animals and reduce potential harm wherever feasible. This project was approved by the Animal Ethics Committee of Suzhou TCM Hospital Affiliated to Nanjing University of Chinese Medicine, Jiangsu Province, China (No: 2020-Lun Research Approval-077).

### 2.3 Preparation of Medicated Serum

The dosage for the rats was calculated by adjusting the human dosage (0.5 g, t.i.d.) according to surface area ratios. The low-dose group (675 mg/kg, t.i.d.) received the equivalent of the standard clinical dose, while the medium-dose group (2.025 g/kg, t.i.d.) was administered three times the low-dose amount [18]. The rats were fasted for 8 hours prior to dose administration. This feeding routine was maintained for three consecutive days. On the fourth day, one hour after the last dose, rats were anesthetized with phenobarbital (50mg/kg, i.p.). Blood samples were collected from the abdominal aorta of the rats. These samples were left to coagulate for one hour before being centrifuged. Subsequently, the serum was extracted and stored at -80°C. The serum samples were then classified into extremely low, low, and medium concentration categories. The extremely low concentration serum samples were diluted two-fold from the low samples, while the low and medium concentration samples were used without dilution.

### 2.4 Cell Source and Treatment

The human tumorous thyroid cell line and papillary thyroid carcinoma (PTC, thyroid papillary carcinoma-1, TPC-1) cell line, provided by the Key Laboratory of Goiter at Jiangsu Provincial Hospital, were utilized in this study. The culture medium was comprised of 1% non-essential amino acids, 1% sodium pyruvate, 10% fetal bovine serum, and 88% RPMI-1640, all stored at 4°C. For reviving the cells, they were quickly thawed in a 37°C water bath, followed by the addition of a small amount of medium to the vial. The cells were then transferred to a 15 mL centrifuge tube, centrifuged, and the supernatant was discarded. After rinsing, the cell pellet was resuspended in the medium and placed in a cell culture incubator. The following day, cell morphology was evaluated, with routine medium changes conducted, with daily microscopic inspections to monitor cell growth and condition.

### 2.5 CCK8 Assay

For experimental preparation, the cell suspension was adjusted to a suitable concentration and 50 µl of the suspension (around 1,000 cells) was added to each well of a 96-well plate. The total volume in each well was then brought to 100 µL with the addition of culture medium. Once the cells reached 70%-80% confluency, drug treatment was applied. The wells were then supplemented with complete medium, extremely low serum, low serum, and medium serum, respectively, and incubated for an additional 24 hours. Following treatment, 10 µL of CCK-8 solution was added to each well. The plate was then incubated at 37°C for 1 hour. Absorbance at 450 nm was measured using a microplate reader, with the absorbance value being directly proportional to the number of viable cells.

### 2.6 Flow Cytometry Analysis

#### 2.6.1 Flow Cytometry Analysis of Cell Cycle

For experimental preparation, the cell suspension was adjusted to a suitable concentration and 200 µl of the suspension (3x10^5^/cm^2^) was placed in each well of a six-well plate, with the total volume brought up to 2 ml using the culture medium. This setup was incubated in a specialized culture box for 24 hours. Once the cells reached 70%-80% confluency, drug treatment was applied. The wells were then supplemented with complete medium, extremely low serum, low serum, and medium serum, respectively, and incubated for an additional 24 hours. Post-incubation, the cells were harvested, centrifuged, washed, and the supernatant discarded. The cell pellet was subsequently treated with 1 ml of DNA staining solution and 10 µl of permeabilization solution, thoroughly mixed, and incubated for 30 minutes before analysis.

#### 2.6.2 Flow Cytometry Analysis of Cell Apoptosis

The cell suspension’s concentration was adjusted, and cells were resuspended in 3 ml of medium. Then, 200 µl of this suspension was allocated to each well of a six-well plate, with the final volume in each well increased to 2 mL using culture medium. The plate was incubated in a specialized culture chamber for 24 hours. Once cell coverage reached approximately 70%-80%, drug treatment was initiated. The culture was then diversified with additions of complete medium, extremely low serum, low serum, and medium serum into the wells, and incubated for a further 24 hours. Subsequently, cells were treated with trypsin (without ethylene diamine tetra-acetic acid), centrifuged, and washed twice to remove the supernatant. The cells were then suspended in an apoptosis-positive control solution, incubated on ice for 30 minutes, and centrifuged to discard the supernatant. After resuspension in precooled buffer, an equal volume of untreated live cells was added to the suspension to achieve the desired cell concentration, and the mixture was evenly distributed into three tubes. The first tube served as a blank control, while the other two underwent treatment with two distinct fluorescent dyes. The samples were incubated at room temperature, protected from light, for 5 minutes. Flow cytometry was performed using adjusted forward scatter (FSC) and side scatter (SSC) settings. Following cytometry, the cells were centrifuged, rinsed with precooled buffer, resuspended, and incubated again with fluorescent dye at room temperature in the dark for 5 minutes. Finally, the apoptosis rate was calculated.

### 2.7 Western Blot Assay

Protein extraction was performed for each experimental group, followed by quantification. The proteins were then separated using electrophoresis and transferred onto membranes, which were subsequently blocked with skim milk. The membranes were incubated overnight at 4°C with primary antibodies targeting IL-6, JAK2, and STAT3. Afterward, secondary antibodies were applied, and the membranes were incubated for an additional 2 hours. Enhanced chemiluminescence (ECL) was used for detection, and the expression levels of the proteins were analyzed, with GAPDH acting as the internal reference.

### 2.8 Statistical Analysis

Statistical analyses were conducted using SPSS version 28.0 (IBM Corp., Armonk, NY), and results are reported as mean ± standard deviation. For comparative analyses, one-way or two-way ANOVA was employed, followed by Tukey’s post hoc test when applicable. A *P* value < 0.05 was considered to be statistically significant.

## 3 RESULTS

### 3.1 The Influence of Sera Containing Different Concentrations of NXP on the Viability of Cells After Different Exposure Times

#### 3.1.1 Effects of NXP Sera on PTC Cells

**Fig. (1):**
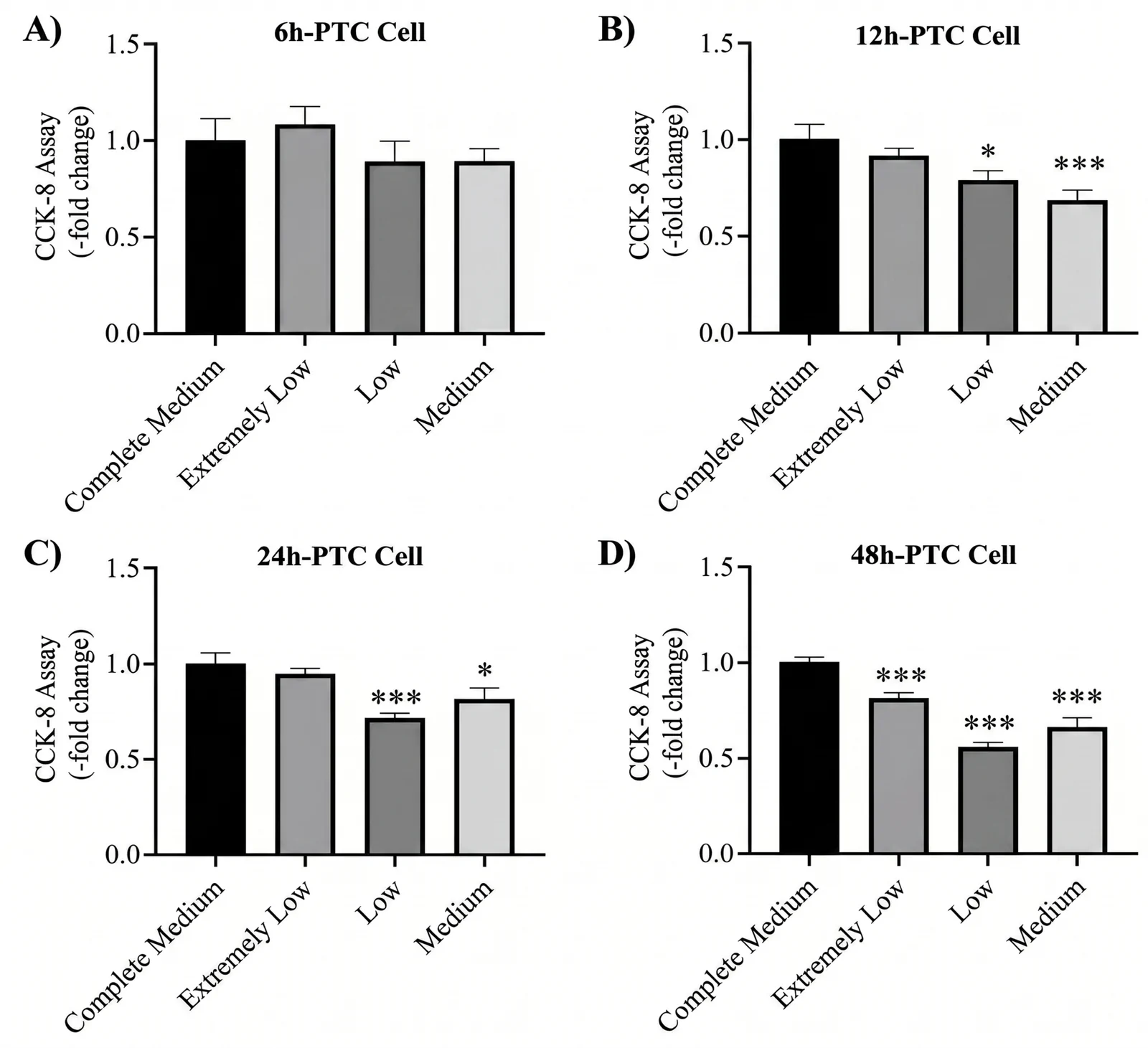
Effects of different concentrations of NXP sera on PTC viability for different exposure times. (**A**) 6-hour exposure time for different concentrations of NXP sera; (**B**) 12-hour exposure time for different concentrations of NXP sera; (**C**) 24-hour exposure time for different concentrations of NXP sera; (**D**) 48-hour exposure time for different concentrations of NXP sera; *P < 0.05, *** *P* < 0.001 *vs.* complete medium (n = 4).

(Fig. **1**) illustrates that after 6 hours of exposure, different NXP concentrations did not notably impact the viability of PTC cells (*P* > 0.05). However, after 12 and 24 hours, sera with low (0.791 ± 0.183-fold change, 0.716 ± 0.048-fold change, respectively) and medium (0.688 ± 0.091-fold change, 0.816 ± 0.100-fold change, respectively) NXP concentrations were found to suppress the viability of these carcinoma cells significantly (*P* < 0.05 *vs*. complete medium group at each time point). Following 48 hours of exposure, all tested NXP concentrations markedly reduced cell viability (0.813 ± 0.050-fold change for extremely low, 0.556 ± 0.043-fold change for low, 0.661 ± 0.085-fold change for medium, *P* < 0.001 *vs.* complete medium).

#### 3.1.2 Effects of NXP Sera on Tumorous Thyroid Cells

As shown in Fig. (**2**), after 6 hours, all concentrations of NXP inhibited the viability of tumorous thyroid cells (0.663 ± 0.299-fold change for extremely low, 0.708 ± 0.088-fold change for low, 0.685 ± 0.092-fold change for medium, *P* < 0.05 *vs*. complete medium). After 12 and 24 hours, both low (0.860 ± 0.143-fold change) and medium (0.805 ± 0.045-fold change) concentrations of NXP demonstrated inhibitory effects on the viability of these cells (*P* < 0.05 *vs*. complete medium). After 48 hours, the low (0.851 ± 0.133-fold change) concentration of NXP significantly suppressed cellular viability (*P* < 0.05 *vs*. complete medium).

According to the above data, considering the effects of sera containing different concentrations of NXP on the viability of cells, and the convenience of experimental time, it was decided to select the 24-hour point for observation post-medication.

### 3.2 The Influence of Sera Containing Different Concentrations of NXP on the Cell Cycle After 24 Hours of Treatment

#### 3.2.1 The Effects of NXP Sera on PTC Cell Cycle

As depicted in Fig. (**3**), flow cytometry analysis showed that after the treatment of PTC cells with sera containing various concentrations of NXP, no significant changes were observed in the proportion of cells in G1 (53.11 ± 4.006% for extremely low, 53.085 ± 2.447% for low, 56.702 ± 2.646% for medium *vs.* 57.576 ± 9.696% for complete medium), S (28.872 ± 2.983% for extremely low, 30.034 ± 3.482% for low, 27.339 ± 2.451% for medium *vs.* 25.548 ± 9.339% for complete medium, and G2 (18.018 ± 1.142% for extremely low, 16.881 ± 1.542% for low, 15.959 ± 0.930% for medium *vs.* 16.876 ± 1.530% for complete medium) stages (*P* > 0.05), suggesting that NXP has no notable impact on the cell cycle of PTC cells.

#### 3.2.2 The Effects of NXP Sera on Tumorous Thyroid Cell Cycle

As depicted in Fig. (**4**), flow cytometry analysis showed that low (40.103 ± 4.392%) and medium (41.914 ± 2.619%) concentration sera from NXP could reduce the proportion of cells in G1 phase (*vs.* 46.854 ± 0.738% for complete medium, *P* < 0.05). Low (12.084 ± 1.259%) and medium (13.130 ± 0.791%) concentration sera significantly decreased the proportion of cells in G2 phase (*vs.* 18.166 ± 0.554% for complete medium, *P* < 0.01). Treatment of tumorous thyroid cells with the three concentrations of sera notably increased the proportion of cells in S phase (38.337 ± 0.798% for extremely low, 47.812 ± 3.304% for low, 44.958 ± 3.108% for medium *vs*. 34.980 ± 0.763% for complete medium, *P* < 0.01). These findings indicate that sera from different concentrations of NXP can arrest the cells in S phase, halting cell division and impeding their progression. This suggests that the influence of NXP on the cell cycle of tumorous thyroid cells mainly manifests in S-phase arrest.

### 3.3 The Influence of Sera Containing Different Concentrations of NXP on the Apoptosis After 24 Hours of Treatment

#### 3.3.1 The Effects on PTC Cell Apoptosis

**Fig. (5):**
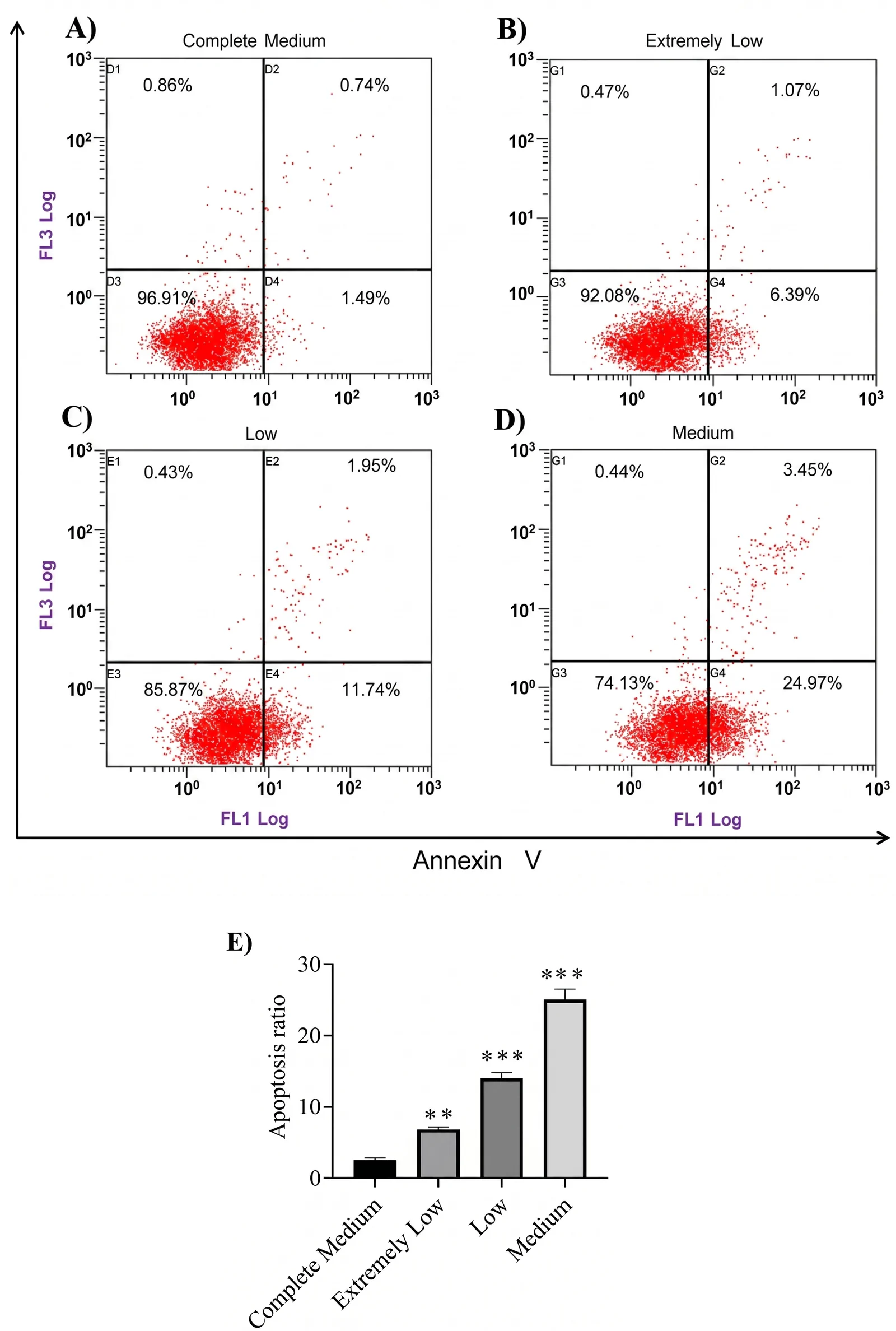
Effects of different concentrations of NXP sera on PTC cell apoptosis after 24 hours of treatment. The apoptosis ratio was determined by using flow cytometry. (**A**) Representative image for the effect of complete medium on the percentage of cell apoptosis. (**B**) Representative image for the effect of extremely low medium on the percentage of cell apoptosis. (**C**) Representative image for the effect of low medium on the percentage of cell apoptosis. (**D**) Representative image for the effect of medium concentrations of NXP sera on the percentage of cell apoptosis. (**E**) Statistical analysis for cell apoptosis ratio in response to 24 hours of NXP sera treatment (n = 4). **P < 0.01, ***P < 0.001 *vs.* complete medium.

Compared to the control group (Fig. **5A**), sera from different concentrations of NXP markedly promoted the apoptosis of PTC cells, and the rate increased with the concentration (6.837 ± 0.739% for extremely low, 13.99 ± 1.984% for low, 25.007 ± 3.697% for medium *vs*. 2.487 ± 0.778% for complete medium, *P* < 0.01). It suggests that NXP can induce apoptosis in PTC cells in a dose-dependent manner (Fig. **5**).

#### 3.3.2 The Effects on Tumorous Thyroid Cells

**Fig. (6):**
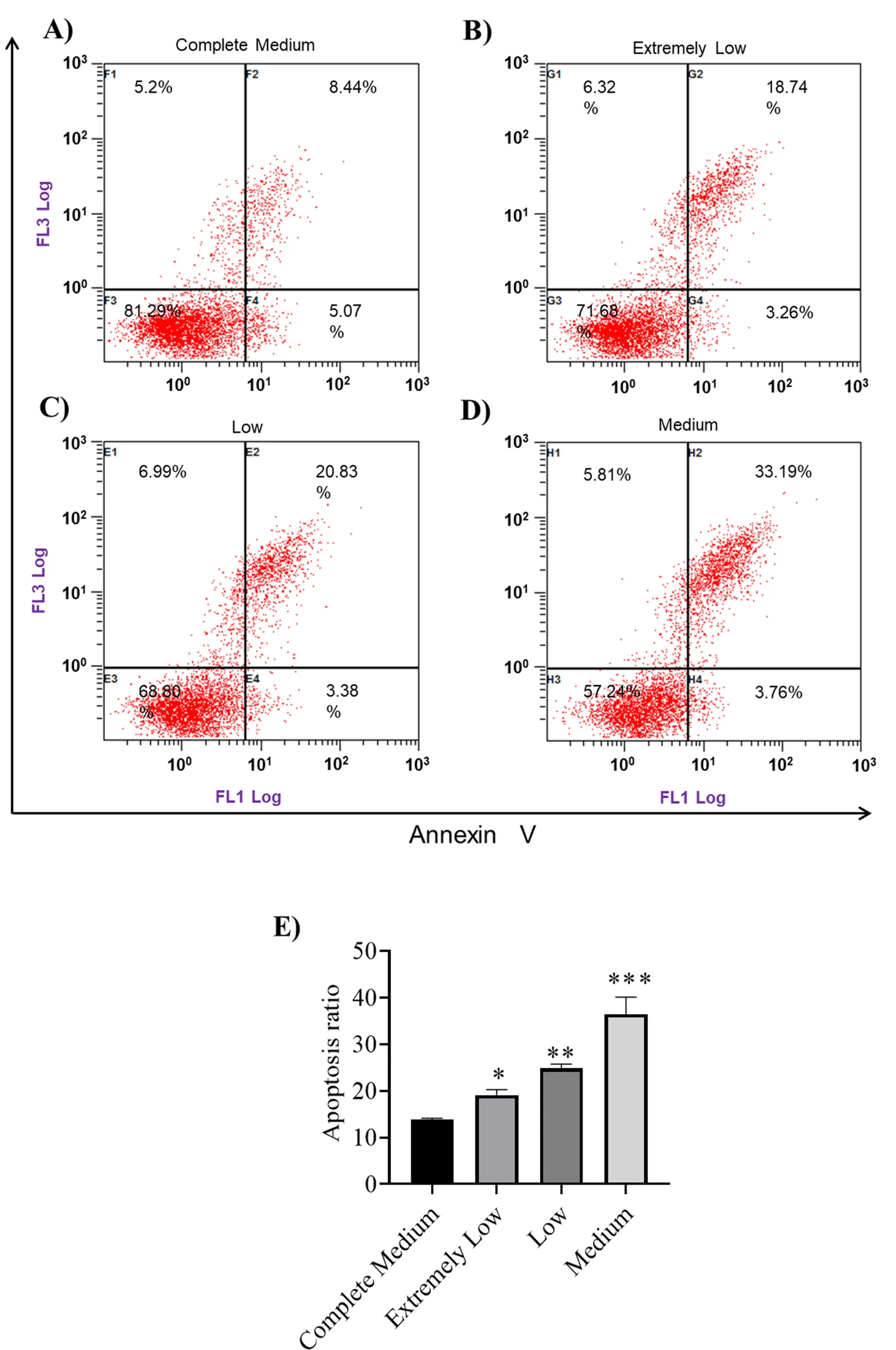
Effects of different concentrations of NXP sera on tumorous thyroid cell apoptosis after 24 hours of treatment. The apoptosis ratio was determined by using flow cytometry. (**A**) Representative image for effect of complete medium on percentage of cell apoptosis. (**B**) Representative image for the effect of extremely low medium on the percentage of cell apoptosis. (**C**) Representative image for the effect of low medium on the percentage of cell apoptosis. (**D**) Representative image for the effect of medium concentrations of NXP sera on the percentage of cell apoptosis. (**E**) statistical analysis for cell apoptosis ratio in response to 24 hours of NXP sera treatment (n = 4). *P < 0.05, **P < 0.01, ***P < 0.001 *vs.* complete medium.

Compared to the control group (Fig. **6A**), sera from different concentrations of NXP could promote the apoptosis of tumorous thyroid cells, and the apoptosis rate relatedly increased with the concentration (19.113 ± 2.846% for extremely low, 24.813 ± 2.247% for low, 36.443 ± 8.991% for medium, *vs*. 13.793 ± 0.832% for complete medium, *P* < 0.05). Moreover, the low concentration of NXP had the most pronounced effect on promoting cell apoptosis (*P* < 0.01). It suggests that NXP can induce the apoptosis of tumorous thyroid cells in a dose-dependent manner (Fig. **6**).

### 3.4 The Influence of Sera Containing Different Concentrations of NXP on the Expression of IL6/JAK2/STAT3 Proteins in PTC Cells After 24 Hours of Treatment

**Fig (7):**
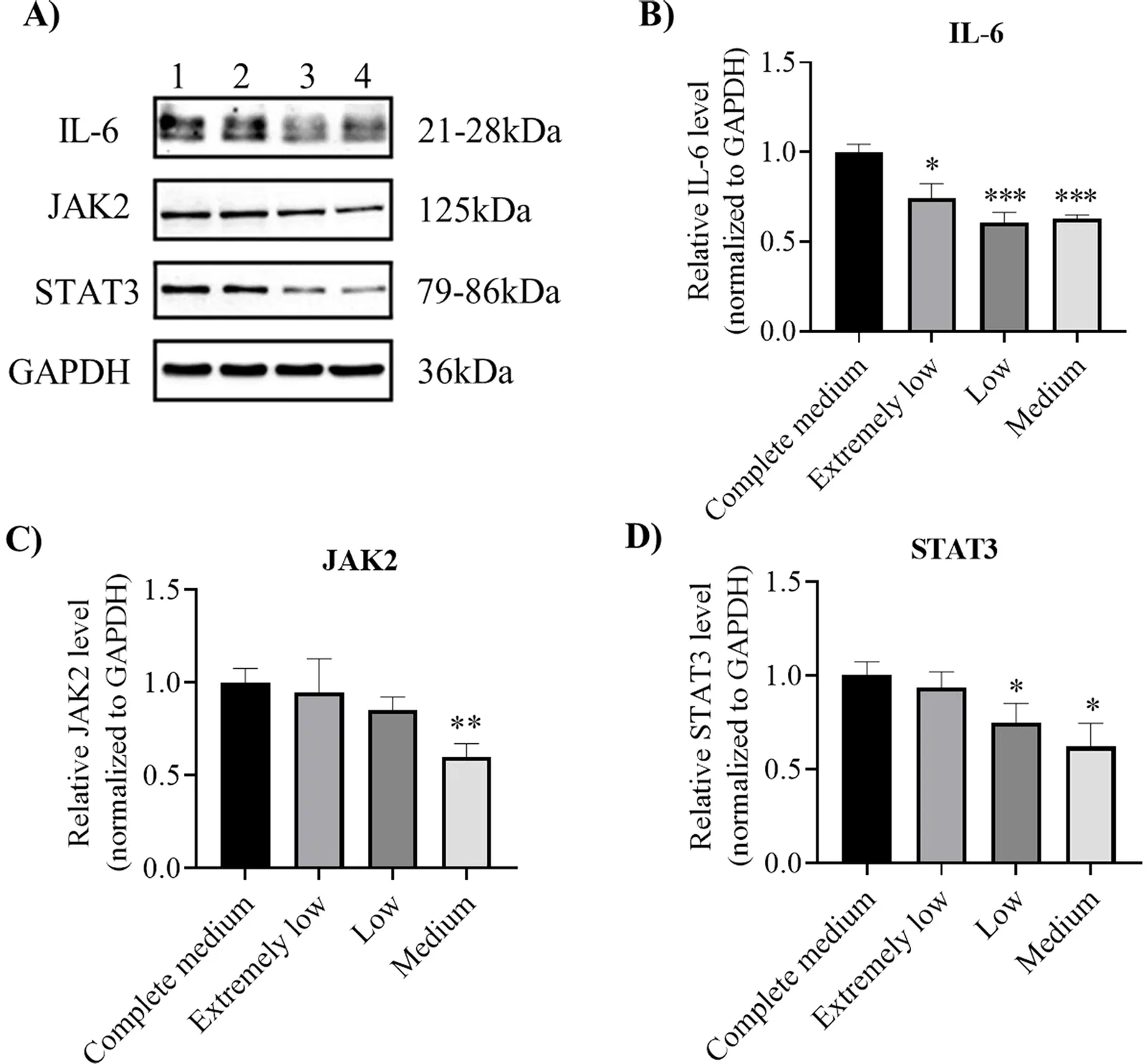
After 24 hours of treatment, sera containing different concentrations of NXP had various influences on the expression levels of IL-6, JAK2, and STAT3 in PTC cells. (**A**) Representative blot of IL-6, JAK2, STAT3 and GAPDH in each group, lane 1 is complete medium, lane 2 is extremely low, lane 3 is low, lane 4 is medium. (**B**) Density analysis of IL-6 expression in each group, (**C**) Density analysis of JAK2 expression in each group, (**D**) Density analysis of STAT3 expression in each group. All data were normalized to the corresponding GAPDH level (n =5). *P < 0.05, ** *P* < 0.01, *** *P* < 0.001 *vs.* complete medium.

Western blot analysis showed that (Fig. **7**), compared to the complete medium group, the medium concentration of NXP could significantly reduce the expression of JAK2 protein (0.598 ± 0.163-fold change *vs.* 1.00 ± 0.169-fold change, *P* < 0.01). Both low (0.747 ± 0.231-fold change) and medium (0.620 ± 0.279-fold change) concentrations of NXP decreased the expression of STAT3 protein (*vs.* 1.00 ± 0.160-fold change, *P* < 0.05), and all different concentrations of NXP reduced IL-6 protein expression (0.743 ± 0.180-fold change for extremely low, 0.609 ± 0.122-fold change for low, 0.628 ± 0.051-fold change for medium, *vs*. 1.00 ± 0.099-fold change for complete medium, *P* < 0.05). The results indicate that NXP can downregulate the expression levels of IL-6, JAK2, and STAT3 proteins in PTC cells to varying degrees, suggesting its possible role in IL6/JAK2/STAT3 signaling pathway.

## 4 DISCUSSION

Regarding the thyroid cytopathology, the Bethesda Classification System is a standardized reporting system for thyroid fine-needle aspiration (FNA) specimens. The 2023 edition classifies FNA pathology reports into six levels: Non-diagnostic (Bethesda I), Benign (Bethesda II), Atypia of Undetermined Significance (Bethesda III), Follicular Neoplasm (Bethesda IV), Suspicious for Malignancy (Bethesda V), and Malignant (Bethesda VI). Bethesda II indicates that there are no signs of malignancy in the thyroid cells, meaning the thyroid nodule is considered benign. The risk of malignancy is very low. In the 2023 edition, the malignancy risk for this category is clearly defined, providing a more accurate reference for clinicians. Bethesda III shows atypical morphology, but it is insufficient to diagnose malignancy. It is a category with ambiguous pathological features, and the cells have some features that deviate from normal cells, but not enough to be classified as malignant. In the 2023 edition, Atypia of Undetermined Significance is divided into two sub-groups based on malignancy risk (ROM) and molecular profiles: Atypia of Undetermined Significance - Nuclear Atypia and Atypia of Undetermined Significance-Other (including architectural atypia, oncocytic atypia, and lymphocytic atypia). The ROM of the former is significantly higher (59%), while that of architectural atypia or oncocytic atypia is about 6.5% [19, 20].

Thyroid nodules (TNs) are growths resulting from abnormal accumulation and proliferation of thyroid tissue, the exact cause of which remains largely unknown. Although most nodules are benign, some may progress to thyroid cancer due to unchecked proliferation of atypical epithelial cells. Global epidemiological data [21] indicate that the incidence of thyroid nodules is increasing annually, driven by socio-economic development and lifestyle shifts, with malignant nodules accounting for 7% to 15% of cases [22]. Initially, TNs may be asymptomatic and are often first identified during routine medical examinations. As they progress, they can impact both appearance and quality of life, prompting individuals to seek interventions like surgical removal or radiofrequency ablation. There is ongoing controversy regarding the optimal surgical treatment for differentiated thyroid carcinoma (TC); total thyroidectomy (TT) and subtotal thyroidectomy (STT) remain the two primary surgical procedures. Researchers have demonstrated that TT can be safely performed in patients with differentiated thyroid carcinoma without increasing the risk of early complications [23]. While these approaches may offer immediate relief, they are associated with potential long-term complications, including thyroid dysfunction, voice alteration, nodule recurrence, and more. Postoperative patients may require lifelong thyroid hormone replacement, imposing additional psychological and financial burdens. Therefore, it is crucial to explore new therapeutic targets, with Traditional Chinese Medicine (TCM) offering promise for its anti-tumor properties and potential as an adjunct treatment. NXP, developed from renowned TCM formulas by practitioners at Suzhou Hospital of Traditional Chinese Medicine, has been shown through years of clinical practice to improve patients’ overall health, reduce TN size, and slow disease progression.

Thyroid nodules (TNs) include both benign growths, primarily thyroid adenomas, and thyroid cancers. In recent years, there has been a noticeable trend of thyroid cancer affecting younger individuals. By 2020, there were approximately 586,000 new cases of thyroid cancer globally, with Papillary Thyroid Carcinoma (PTC) accounting for up to 90% of these cases [22], making it the most prevalent histological variant. In this study, to thoroughly investigate the distinct biological characteristics of benign and malignant nodules, we selected both tumorous thyroid cells and PTC cells for examination. Our findings indicate that serum with a specific concentration of NXP not only decreases the viability of both tumorous thyroid cells and PTC cells but also suppresses the proliferation of tumorous thyroid cells and triggers their apoptosis. Additionally, although NXP-containing serum did not significantly impact the cell cycle of PTC cells, it was capable of inducing apoptosis in a dose-dependent manner. These findings suggest potential mechanisms by which NXP may reduce nodule size and decelerate the progression of the disease.

Apoptosis, the most extensively investigated form of programmed cell death, plays a vital role in various biological functions, including the maintenance of adult tissue homeostasis and developmental processes, as well as the selective elimination of abnormal cells in both physiological and pathological contexts. Disruptions in apoptotic processes can trigger various diseases [24]. In relation to thyroid health, the balance between cell proliferation and apoptosis is essential for normal thyroid function. An imbalance, characterized by excessive cellular proliferation or inhibited apoptosis, can result in abnormal tissue enlargement, ultimately leading to nodule formation [25]. Therefore, restoring the balance between proliferation and apoptosis of thyroid cells is a key therapeutic objective in managing thyroid nodules. Our current findings suggest that NXP may help restore this balance in thyroid cells.

The development of tumors is a complex, multi-step process influenced by numerous cellular factors. Within this context, the tumor inflammatory microenvironment, driven by various components, notably facilitates tumor progression. Interleukin-6 (IL-6), a versatile cytokine produced by activated T cells and fibroblasts, plays a pivotal role not only in cell differentiation and proliferation but also in immune regulation, host defense, and inflammatory responses [26]. It directly stimulates tumor cell proliferation, migration, and invasion [27, 28]. Research has shown that IL-6 is instrumental in the advancement of various cancers, including those of the breast, ovary, lung, prostate, and liver cancers [29-33]. Additionally, tumor-associated macrophages amplify the oncogenic potential and drug resistance of cancer stem cells through the secretion of IL-6 [34]. IL-6 mediates its biological effects primarily through three signaling pathways: the JAK/STAT pathway, the Ras/ERK pathway, and the PI3K-mediated pathway [35, 36]. The JAK/STAT pathway involves JAK, a protein tyrosine kinase, and STAT proteins, which act as signal transducers and transcription activators. When cytokine receptors engage with their ligands, STAT proteins are activated, translocate to the nucleus, and regulate gene expression to elicit specific biological outcomes. The JAK2/STAT3 pathway is predominantly activated, where intracellular JAK2 is phosphorylated upon binding to its receptor through the IL-6 specific receptor, leading to further phosphorylation of STAT3 [37]. This phosphorylated STAT3, utilizing its DNA binding domain, acts as a transcription factor to modulate the expression of various downstream target genes [38], thereby influencing tumor proliferation, apoptosis, and promoting malignant cell transformation [39].

In recent years, the role of the IL-6/JAK2/STAT3 signaling pathway in tumor initiation has emerged as a significant focus of research. This pathway is known to be abnormally overactive in various cancers, significantly impairing the immune system’s ability to combat tumors [40]. Studies have revealed that in papillary thyroid carcinoma (PTC), the expression of ANXA1 is notably elevated, correlating with tumor growth and poor outcomes for patients. Through techniques such as bioinformatics analysis, RT-PCR, and immunohistochemistry, Zhao *et al*. have shown that ANXA1 overexpression in PTC cells enhances IL-6 production, leading to increased phosphorylation of JAK2 and STAT3 [41]. This suggests ANXA1’s pivotal role in regulating the malignancy of PTC through the IL-6/JAK2/STAT3 pathway, enhancing cell proliferation and metastasis both *in vitro* and *in vivo*. Chen *et al*. [42] reported that the traditional Chinese medicine formula Jianpi Yangzheng Xiaozheng Decoction (JPYZXZ) could reduce the expression of proteins JAK2, STAT3, IL-6, and phosphorylated STAT3, as well as PDL1 levels in gastric cancer cells. This indicates JPYZXZ has the potential to diminish PD-L1 levels by targeting the IL-6/JAK2/STAT3 pathway upstream, reducing cytokine accumulation that mediates immune suppression and inflammation. This could help remodel the immunosuppressive tumor microenvironment and potentially slowing tumor progression. Additionally, Sun *et al*. [43] discovered that 2-hydroxy-3methylanthraquinone (HMA), a compound extracted from Hedyotis diffusa, significantly inhibits the growth and invasion of A549 lung cancer cells induced by IL-6, a process associated with increased cellular apoptosis and deactivation of the IL-6/JAK2/STAT3 pathway. Earlier research also supports the idea that blocking this pathway can reduce the tumor-forming capacity of A549 and CL1-5 cells [44]. These findings highlight the potential of targeting the IL-6/JAK2/STAT3 signaling pathway and its components as a strategic approach for cancer prevention and therapy.

Our study found that, in comparison to the control group, the medicated serum derived from NXP led to a notable reduction in the levels of IL-6, JAK2, and STAT3 proteins in PTC cells. This suggests that NXP could play a role in modulating tumor cell proliferation and apoptosis by targeting the IL-6/JAK2/STAT3 signaling pathway. Such an intervention may influence the tumor’s inflammatory microenvironment, potentially controlling tumor growth or even causing tumor shrinkage, thereby delaying disease progression and offering a novel therapeutic strategy for thyroid neoplasia through pathway inhibition. However, the current understanding of NXP’s mechanistic effects on thyroid neoplasia is primarily based on inflammatory marker assessments, highlighting a gap in comprehensive exploration and representing a potential limitation of this study. Moving forward, we aim to investigate the functional components and underlying mechanisms of NXP’s effects more thoroughly, in order to provide a stronger scientific foundation for its clinical use in traditional Chinese medicine.

A limitation of the present study is that the data are obtained from the cell-based observation, lacking in-vivo validation. In addition, although NXP is a traditional Chinese patent medicine, the current study only explored its impact on the IL6/JAK2/STAT3 signaling pathway. TCM usually contains multiple active ingredients, and these ingredients may act on multiple targets and pathways simultaneously. The study did not comprehensively analyze the complex interactions between different components of NXP and various biological processes, which may lead to an incomplete understanding of the overall mechanism of NXP. As mentioned in the background, due to the complex nature of TCM, there is limited clinical research on NXP. The current in-vitro study lacks strong support from clinical data. Clinical trials are needed to verify the safety and efficacy of NXP in patients with TN, including aspects such as appropriate dosage, treatment duration, and potential adverse reactions. Future research should focus on conducting in-vivo animal experiments using TN-induced animal models to observe the effect of NXP on TN development. Omics-based technologies such as transcriptomics, proteomics, and metabolomics can be used to comprehensively analyze the changes in gene expression, protein levels, and metabolite profiles in cells or tissues treated with NXP. Importantly, well-designed clinical trials are essential to evaluate the real-world effectiveness and safety of NXP in treating TN patients.

## CONCLUSION

The present findings suggest that NXP may exert anti-tumorigenic effects by inhibiting the viability of tumorous thyroid and papillary thyroid carcinoma (PTC) cells, while simultaneously modulating the equilibrium between cell proliferation and apoptosis. This observed effect appears to be mediated through the modulation of the IL-6/JAK2/STAT3 signaling pathway, which in turn influences the expression of associated inflammatory cytokines. These insights into NXP’s mechanism of action provide a rationale for exploring its potential as a therapeutic strategy in the management of thyroid nodules (TN), particularly in slowing disease progression. The results highlight the importance of targeting inflammatory signaling pathways in thyroid malignancies, offering a foundation for further investigation into NXP’s therapeutic potential in this context.

## AUTHORS’ CONTRIBUTIONS

The authors confirm their contributions to the paper as follows. YY was responsible for data collection. TC performed the analysis and interpretation of the results. XY and FH contributed to drafting the manuscript. YCW validated the findings, while WW handled data curation. WTM developed the methodology, and YTZ conducted the investigation. All authors reviewed the results and approved the final version of the manuscript.

## Figures and Tables

**Fig. (2):**
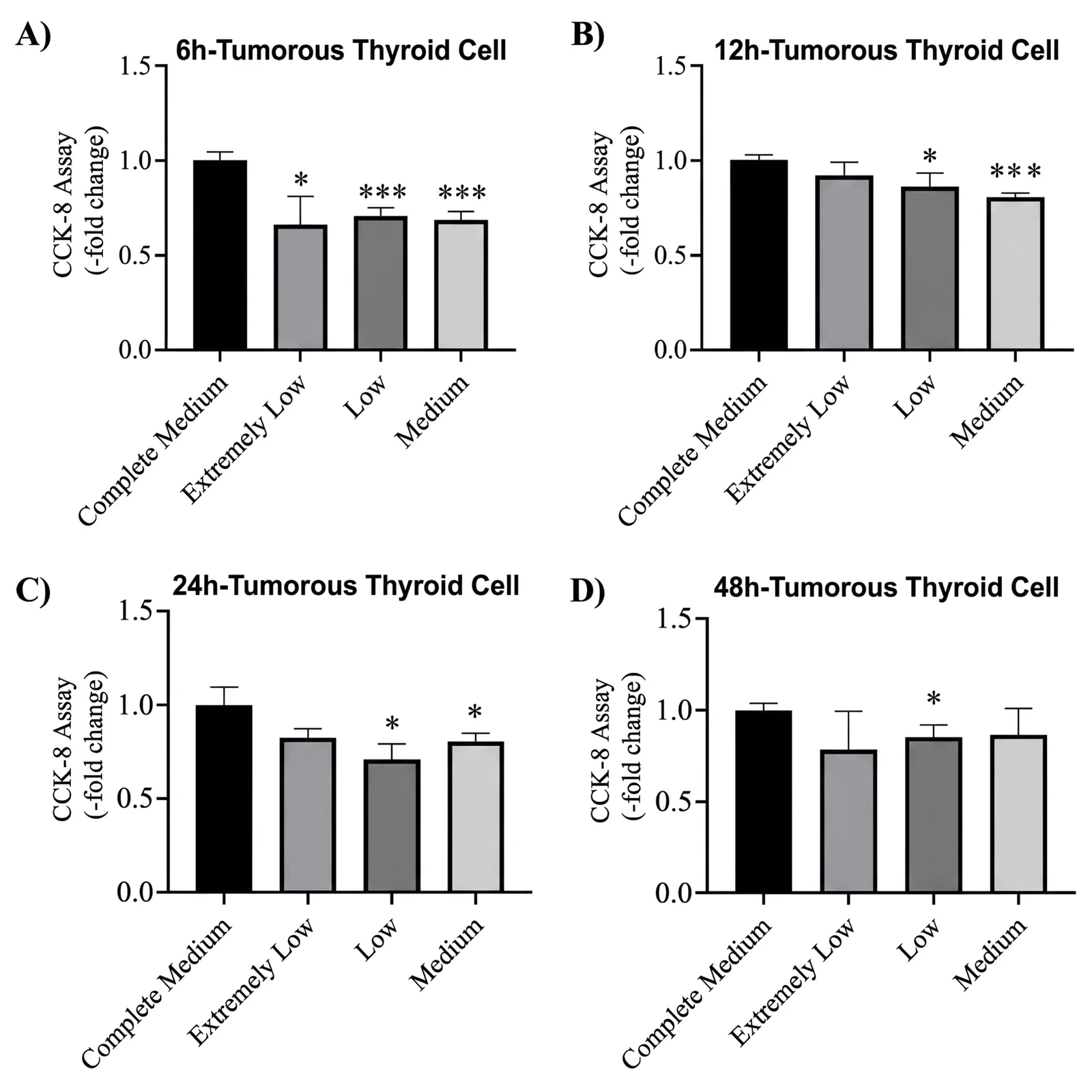
Effects of different concentrations of NXP sera on tumorous thyroid cell viability for different exposure times. (**A**) 6-hour exposure time for different concentrations of NXP sera; (**B**) 12-hour exposure time for different concentrations of NXP sera; (**C**) 24-hour exposure time for different concentrations of NXP sera; (**D**) 48-hour exposure time for different concentrations of NXP sera; *P < 0.05, *** *P* < 0.001 *vs.* complete medium (n = 4).

**Fig. (3):**
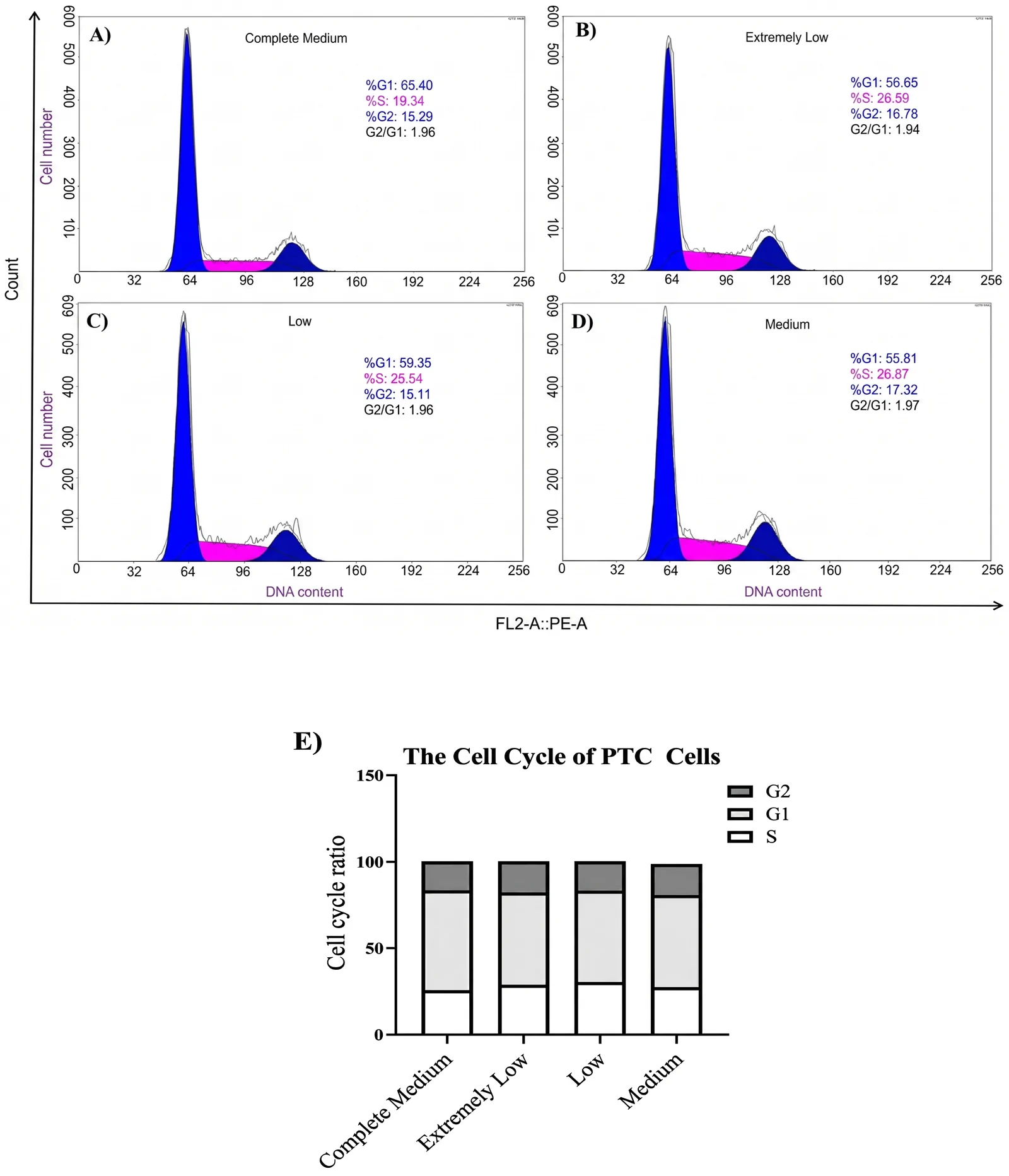
Effects of different concentrations of NXP sera on PTC cell cycle after 24 hours of treatment. The proportion of cells in different phases was determined by using flow cytometry. (**A**) Representative image for the effect of complete medium on the percentage of cell stages. (**B**) Representative image for the effect of extremely low medium on the percentage of cell stages. (**C**) Representative image for the effect of low and medium on the percentage of cell stages. (**D**) Representative image for the effect of medium on the percentage of cell stages. (**E**) statistical analysis for cell cycle ratio at different stages in response to 24 hours of NXP sera treatment (n = 4).

**Fig. (4):**
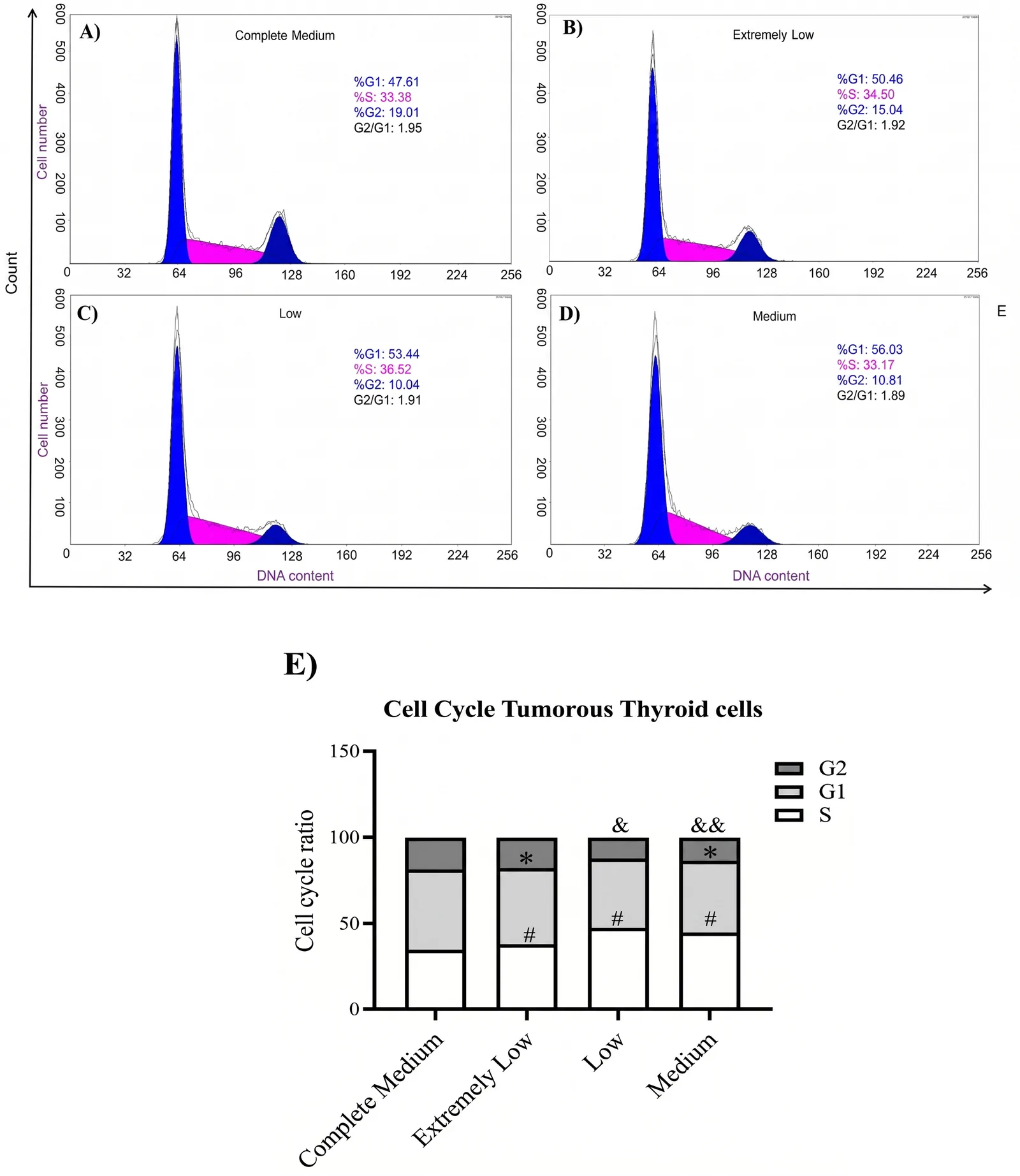
Effects of different concentrations of NXP sera on the tumorous cell cycle after 24 hours of treatment. The proportion of cells in different phases was determined by using flow cytometry. (**A**) Representative image for the effect of complete medium on the percentage of cell stages. (**B**) Representative image for the effect of extremely low medium on the percentage of cell stages. (**C**) Representative image for the effect of low medium on the percentage of cell stages. (**D**) Representative image for the effect of medium concentrations of NXP sera on the percentage of cell stages. (**E**) Statistical analysis for cell cycle ratio at different stages in response to 24 hours of NXP sera treatment (n = 4). *P < 0.05, ^#^ *P* < 0.01, ^&^ *P* < 0.01, ^&&^ *P* < 0.001 *vs.* complete medium.

## Data Availability

The authors confirm that the data supporting the findings of this study are available within the article.

## LIST OF ABBREVIATIONS

TN: Thyroid Nodules
TCM: Traditional Chinese Medicine
NXP: Nei-Xiao-Pian
IL-6: Interleukin-6
JAK2: Janus Kinase 2
STAT3: Signal Transducer and Activator of Transcription 3
PTC: Papillary Thyroid Carcinoma
JPYZXZ: Jianpi Yangzheng Xiaozheng Decoction
